# Chiropteran diversity in the peripheral areas of the Maduru-Oya National Park in Sri Lanka: insights for conservation and management

**DOI:** 10.3897/zookeys.784.25562

**Published:** 2018-09-12

**Authors:** Gayan Edirisinghe, Thilina Surasinghe, Dinesh Gabadage, Madhava Botejue, Kalika Perera, Majintha Madawala, Devaka Weerakoon, Suranjan Karunarathna

**Affiliations:** 1 Wild Rescue Team, 183/6, Horana Road, Kesbewa, Piliyandala 10300, Sri Lanka Biodiversity Conservation Society Nugegoda Sri Lanka; 2 Department of Biological Sciences, Bridgewater State University, Bridgewater, MA, USA Wild Rescue Team Piliyandala Sri Lanka; 3 Biodiversity Conservation Society, No: 150/6, Stanly Thilakaratne Mawatha, Nugegoda 10250, Sri Lanka Bridgewater State University Bridgewater United States of America; 4 Central Environmental Authority, No 104, Denzil Kobbekaduwa Mawatha, Battaramulla 10120, Sri Lanka Central Environmental Authority Battaramulla Sri Lanka; 5 No: 95/2, Subadhrarama Road, Nugegoda 10250, Sri Lanka Unaffiliated Nugegoda Sri Lanka; 6 South Australian Herpetology Group, South Australian Museum, North Terrace, Adelaide, SA 5000, Australia Nature Explorations & Education Team Moratuwa Sri Lanka; 7 Department of Zoology, University of Colombo, Colombo 03, 00300, Sri Lanka South Australian Museum Adelaide Australia; 8 Nature Explorations & Education Team, No. B-1/G-6, De Soysapura Flats, Moratuwa 10400, Sri Lanka University of Colombo Colombo Sri Lanka

**Keywords:** behavior, conservation, habitat associations, roosting sites, species richness, threats

## Abstract

In Sri Lanka, there are 31 species of bats distributed from lowlands to mountains. To document bat diversity and their habitat associations, 58 roosting sites in Maduru-Oya National Park periphery were surveyed. Fifteen bat species were recorded occupying 16 different roosting sites in this area. Among all the species recorded, *Rhinolophusrouxii* was the most abundant species per roosting site whereas *Kerivoulapicta* was the least abundant. A road-kill specimen similar to genus *Phoniscus* was found during the survey, a genus so far only documented in Southeast Asia and Australasia. Although our study area provided habitats for a diverse chiropteran community, the colony size per roost was remarkably low. Although our study area is supposedly a part of the park’s buffer zone, many anthropogenic activities are threatening the bat community: felling large trees, slash-and-burn agriculture, excessive use of agrochemicals, vengeful killing, and subsidized predation. We strongly recommend adoption of wildlife-friendly sustainable land management practices in the buffer zone such as forest gardening, agroforestry (alley cropping, mixed-cropping), and integrated farming. Bat conservation in this region should take a landscape-scale conservation approach which includes Maduru-Oya National Park and other surrounding protected areas into a regional conservation network. Extents of undisturbed wilderness are dramatically declining in Sri Lanka; thus, future conservation efforts must be retrofitted into anthropocentric multiuse landscapes and novel ecosystems like areas surrounding Maduru-Oya National Park.

## Introduction

Sri Lanka is a small (65,610 km^2^), Indian Oceanic tropical island providing habitats for a rich mammalian diversity containing 126 species including 23 (~18%) endemic species (de Silva Wijeyeratne 2016; Leowinata and Luk 2016; Ministry of Environment 2012). The island provides habitats for a number of charismatic mega-mammals, such as, the Asian elephant, Sri Lankan leopard, sloth bear, and Old-World monkeys (de Silva Wijeyeratne 2014; Weerakoon and Goonatilake 2006). The majority of Sri Lanka’s mammalian diversity is comprised of small and medium-sized mammals, including 31 species of chiropterans that belongs to 14 yinpterochiropteran and 18 yangochiropteran species (Leowinata and Luk 2016; Yapa 2017). Currently, 18 (58%) Sri Lankan bat species are “nationally threatened” (five Critically Endangered, five Endangered, and eight Vulnerable) (Ministry of Environment 2012). As evident from the southeast-Asian island Singapore where 72% of chiropterans have gone extinct, island bats are more vulnerable to anthropogenic stressors and habitat loss (Lane et al. 2006; Mickleburgh et al. 2002). Due to greater access to roosts and diversified food niches, woodlands and forests are the primary habitats for most bats, although a handful of synanthropic species inhabit built-up urban environments (Avila-Flores and Fenton 2005; Gehrt and Chelsvig 2003). Bats are among the most imperiled mammals, particularly in the tropical realm including Sri Lanka, due to decline in prey availability, pesticide use, roost destruction, and deforestation (Mickleburgh et al. 2002; Ministry of Environment 2012).

Availability and diversity of roosting sites are paramount elements of life history of bats. A number of critical biological functions, such as reproduction, postnatal care, predator avoidance, and thermoregulation are provisioned by roosting sites (Campbell et al. 2006; Chaverri and Kunz 2010; Kunz and Lumsden 2003; Lewis 1995). Although bats in general are known to occupy a diverse range of habitats for roosting, studies conducted in Sri Lanka have mostly reported caves as suitable roosting sites (Kusuminda et al. 2013; Rubsamen et al. 2004; Yapa and Ratnasooriya 2006; Yapa 1992). Although behavior, echolocation, and trophic ecology of bats have been satisfactorily explored in Sri Lanka (Neuweiler et al. 1987; Pavey et al. 2001; Schmidt et al. 2011), much remain unknown about selection of roosting sites and habitat associations. Thus, surveying roosting sites helps understanding habitat associations of bats in areas of interest.

There is a pressing need for local inventories of bat diversity to map species distributions throughout the island, which is critical for conservation assessments. Amongst the remaining Sri Lankan forest cover, the dry-mixed evergreen forests are largely secondary in origin, the most extensive and embedded in rural-agricultural landscapes (covering 21% of the island’s land area) (FAO 2010a; b); yet these habitats remained relatively unexplored in terms of bat diversity. Therefore, documenting bat diversity in less-explored habitats is crucial for updating bat distribution ranges and to inform conservation planning. Although conservation lands provide substantial immunity against habitat degradation, bat communities occupying outside protected areas could be highly vulnerable to anthropogenic threats. Thus, investigating bat diversity and their use of roosting sites are salient for conservation.

Our specific objectives in this study were to (1) document species richness of bats in the periphery of Maduru-Oya National Park; (2) investigate their roosting site selection and identity local and landscape-scale land-cover types that influence presence of bats in potential roosting sites. Surveying habitats outside conservation lands is of conservation importance for several reasons. First, species distribution ranges may not be restricted by conventional protected area boundaries, thus bat occupancy can extend into the peripheral habitats of Maduru-Oya National Park. Thus, field surveys are necessary to confirm species occupancy outside the park. Secondly, we attempted to identify suitable bat habitats outside the park and thereby help re-delineation and management of a park buffer zone. Third, although bat habitats inside the park are protected from human disturbances and habitat loss, the same cannot be said for habitats of the park periphery. Therefore, documenting bat diversity and threats outside the park can help develop wildlife-friendly habitat management practices.

## Materials and methods

### Study area

**Figure 1.**
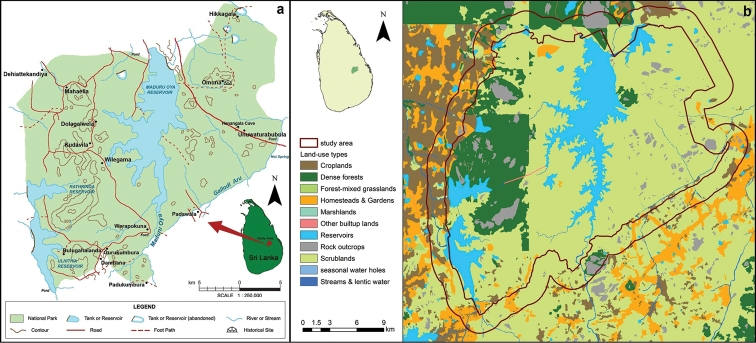
**a** the local topography, road network, local towns and hydrology and **b** land–used types (the 2km-wide study area is delineated by solid lines) in and around Maduru-Oya National Park.

Peripheral landscapes of Maduru-Oya National Park (~58,850 ha; 7.3833–7.5833N and 81.033–81.3333E) comprise diverse habitat mosaics including woodlands, teak plantations, scrublands, grasslands, home gardens, croplands, lakes and reservoirs, streams, marshlands, and a variety of build-up environments (Figure 1). The main vegetation type of the area is tropical dry mixed-evergreen (semi-evergreen) forests (Gunatilleke and Gunatilleke 1990). Teak plantations and unprotected woodlands in these areas are subjected to repeated slash-and-burn agriculture (Gabadage et al. 2015). Located in the dry zone lowlands (annual average precipitation <2,000 mm, annual average temperature 28.7 °C; elevation < 500 m), our study area is influenced by the northeastern monsoon rain (October – late January) (Green 1990; Survey Department of Sri Lanka 2007). Maduru-Oya National Park falls within Mahaweli Development Area, which is a government-sponsored, large-scale socioeconomic development scheme (Gabadage et al. 2015). Reforestation after abandonment of ancient civilizations and slash-and-burn farming practices in our study area have resulted in large extents of secondary forests and scrublands (Pemadasa 1990). The local topography is mostly comprised of lowlands (30–150 m in elevation range), an isolated residual mountain (485 m), an 8 km long vegetated rocky outcrop, and granite caves (Figure 1).

### Field survey

We conducted this survey for 2.5 years (May 2014 to December 2016) and surveyed 58 roosting sites found within a 2 km-wide peripheral area around Maduru-Oya National Park. Surveying roosting sites is highly effective and time-efficient for assessing bat diversity in a given region (Phelps et al. 2016). We surveyed each roosting site both day (0800–1400 h) and night (1900–0100 h) and revisited each roosting site twice in two different years. Based on visual encounter surveys and using headlamps, we searched through a diverse range of roosting sites such as caves (11), rock crevices (4), rocky ledges (2), large tree crowns (5), tree hollows (8), abandoned buildings (8), homesteads (2), culverts (3), bridges (5), banana plantations (6), and paddy fields (4). At each roosting site, we recorded the species found and the relative abundance of each species based on direct roost count. Using an endoscope camera and an 8 mm illuminated camera head (Work zone – China), we documented bat occupancy inside tree cavities. We captured flying bats using hoop-nets (Circumference 2.5 m) and released them at site of capture after identification. We identified bats using published standard keys and guides (Bates and Harrison 1997a; Corbet and Hill 1992; Phillips 1980; Srinivasulu et al. 2010) and photographed with an SLR digital camera (Cannon EOS 60D). In addition to roosting site surveys, we documented road-kill bats on a 10 km stretch of a double lane highway (the major highway found within the park periphery) by walking along the highway on both edges of the road while making visual observations (both on the road as well as the road verges) for bat carcasses. If a road-kill bat was found, we identified the bat to the highest possible taxonomic level. After identification, we moved the carcasses away from the road to avoid secondary road-kills. We repeated the road-kill survey six times in a given year throughout the study period. Our study conforms to the guidelines of American Society of Mammologists; the IACUC committee of Bridgewater State University approved our research procedure.

### Data analyses

To test for differences in species compositions among different roosting sites, we ran a Multiple Response Permutation Procedures (MRPP) treating species composition of bats as response variable and different types of roosting sites as predictor variables. Here, we ran 500 permutations and used Bray-Curtis Index to calculate the distance matrix. To study patterns of roosting site selection by different species of bats, we constructed an ordination based on NMDS (non-metric multidimensional scaling). We calculated the distance measures from Bray Curtis index for different bat species. We extracted two axes with the lowest final stress that best ordinated the roosting sites in species space through 500 iterations with random starting configurations. To select dimensionality with the lowest stress, we ran a Monte Carlo test (60 runs from real data, and 60 runs from randomized data). We constructed an ordination plot with optimal axes to visualize patterns of roosting site selection. We used *R*–Studio (vegan and mgcv packages) for all statistical analyses (*R* Core Team 2017; *R*–Studio Team 2016).

To investigate the influence of land-use and land-cover types around potential roosting sites on presence of bats (using ArcGIS Pro 2.2) we extracted land-use and land-cover data from a reclassified global land cover data (European Space Agency 2017) for a 500 m (local scale) and a 5 km-radius (landscape-scale) around surveyed roosting sites. We classified each of the roosting site into two binary responses, bats present (through direct and indirect evidence) versus bats absent, and constructed a binomial (logit function) generalized additive model (GAM) where the binary response of presence or absence of bats was treated as the response variable and percentage land-cover types at 500 m and 5 km radii around each roosting site as the predictor variables.

## Results

### Species richness of the bat community

**Figure 2.**
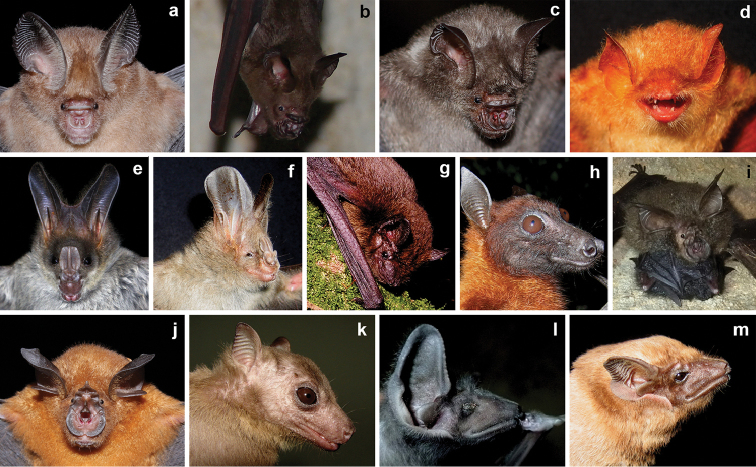
Bat species recorded in the periphery of Maduru-Oya National Park. **a***Hipposiderosater***b***Hipposideroslankadiva***c***Hipposiderosspeoris***d***Kerivoulapicta***e***Megadermalyra***f***Megadermaspasma***g***Pipistrellustenuis***h***Pteropusgiganteus***i***Rhinolophusbeddomei***j***Rhinolophusrouxii***k***Rousettusleschenaulti***l***Taphozouslongimanus***m***Taphozousmelanopogon*.

**Figure 3.**
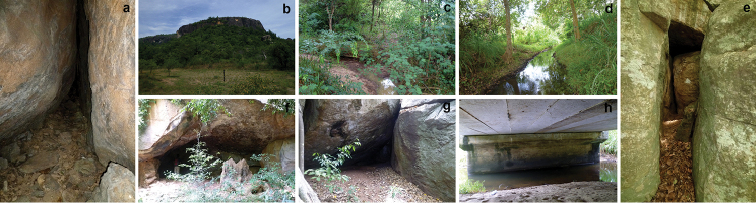
Habitats of bats in the peripheral areas of Maduru-Oya National Park. **a** a cave in Dananjaya Gala **b** rocky outcrops surrounded by forests **c** scrublands with temporary pools **d** small canal inside the forest **e** a cave nearby the Maduru-Oya reservoir **f** a historical cave in Damminna **g** a cave nearby Henanigala **h** under a large bridge.

We recorded a total of 15 species of bats including 10 yinpterochiropteran and five yangochiropteran species representing six chiropteran families (Table 1, Figure 2). The bat species richness we documented around Maduru-Oya National Park accounted for 71% of yinpterochiropteran species and 29% of yangochiropteran species of Sri Lanka (Figure 2). Bats occupied a variety of roosting sites, including the canopy of large trees, tree cavities and hollow trees, abandoned buildings, underneath bridges, caves, and banana plants (Figure 3). Although we surveyed 58 potential roosting sites, only 15 sites had bats. Even though we did not encounter bats in 14 more sites, we documented indirect evidence for bat occupancy– such as characteristic bat odor and presence of fresh guano. We had neither direct nor indirect evidence for bat occupancy in the rest of 29 sites. The roosting sites we surveyed were overwhelmingly used for day roosting. However, *Hipposiderosater*, *Megadermalyra*, and *Hipposideroslankadiva* were only found in their respective roosting sites during night visits. *Rousettusleschenaulti* used the same roosting site both day and night. Among all species, *Rhinolophusrouxii* was, on average, the most abundant species per roosting site (250–300) whereas *Kerivoulapicta* was the least abundant (1–3). All bat species we recorded in this survey were considered “least concerned” in the Global IUCN Red List. In contrast, national conservation assessments of Sri Lanka listed six species “threatened” (one endangered and five vulnerable) and one species “near threatened” (Table 1).

**Table 1.** Roosting sites used by different bat species in the peripheral areas of Maduru-Oya National Park, Sri Lanka and relative abundance of each bat species at each type of roosting site. The number of sightings indicates the number of different days on which each bats species was present at a given roosting site. Superscripts denote national conservation status LC: least concerned, NT: near threatened, VU: vulnerable, EN: endangered. The global conservation status for all species was “least concerned”. Two more species (*Cynopterussphinx* and Phoniscuscf.jagorii) were only recorded as dead specimens.

Family	Species	Trophic guild	Rooting site	Total no. of sightings	Avg. no. of individuals (std. dev.)	Used for day or night roosting?
Pteropodidae	* Pteropus giganteus * ^LC^	Frugivore	Large tree	11	22.54 (4.39)	Both
	* Rousettus leschenaulti * ^LC^	Frugivore	Abandoned building	34	15.60 (1.30)	Both
Hipposideridae	* Hipposideros ater * ^LC^	Insectivore	Abandoned building	10	5.56 (2.45)	Night
	* Hipposideros lankadiva * ^VU^	Insectivore	Underneath bridge	11	4.09 (2.34)	Night
	* Hipposideros speoris * ^LC^	Insectivore	Cave	18	16.90 (0.32)	Both
			Cave	18	29.08 (4.25)	Both
			Cave	18	49.56 (4.12)	Both
Vespertilionidae	* Kerivoula picta * ^NT^	Insectivore	Banana shrub	21	2.13 (0.99)	Day
	* Pipistrellus tenuis * ^LC^	Insectivore	Hollow tree	10	6.78 (4.18)	Both
Megadermatidae	* Megaderma lyra * ^VU^	Carnivore	Underneath bridge	26	28.62 (2.09)	Night
			Underneath bridge	26	22.4 (2.35)	Night
	* Megaderma spasma * ^VU^	Carnivore	Abandoned building	34	66.33 (3.89)	Both
Rhinolophidae	* Rhinolophus beddomei * ^VU^	Insectivore	Cave	09	5.11 (1.69)	Both
	* Rhinolophus rouxii * ^LC^	Insectivore	Abandoned building	34	285.00 (5.50)	Both
Emballonuridae	* Taphozous longimanus * ^EN^	Insectivore	Cave	09	40.14 (3.02)	Both
			Cave	34	9.73 (5.36)	Both
	* Taphozous melanopogon * ^VU^	Insectivore	Cave	15	17.10 (6.51)	Both

**Figure 4.**
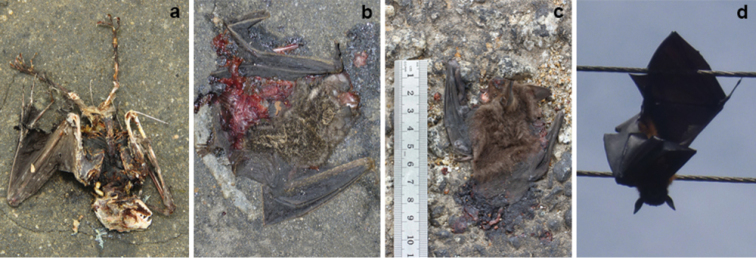
Dead specimens recorded in the peripheral areas of Maduru-Oya National Park **a***Cynopterussphinx***b**Phoniscuscf.jagorii**c***Rhinolophusrouxii***d***Pteropusgiganteus*.

**Figure 5.**
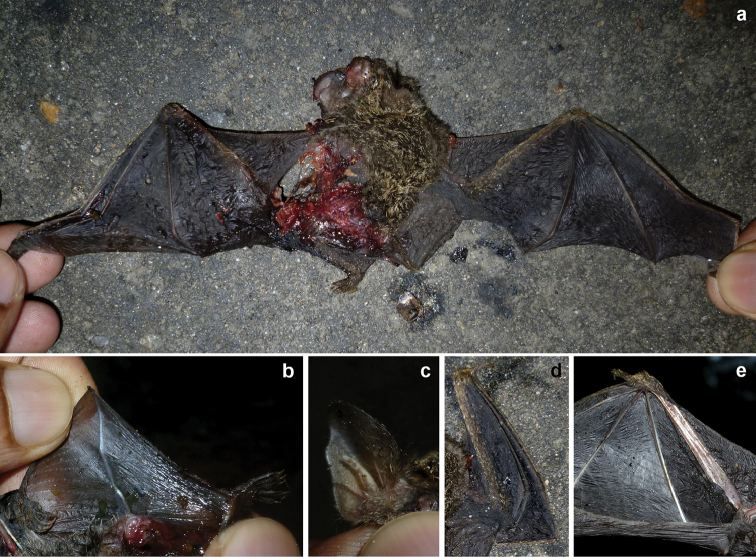
Road killed specimen of Phoniscuscf.jagorii with key characters **a** wingspan with the dorsal body color **b** Interfemoral membrane is well developed and semi–transparent (parts of the interfemoral membrane was damaged) **c** Long, tapering, notched tragus **d** forearm and digits covered with short, golden hair **e** ventral aspect of the wing.

We recorded three bats as road-kills, and identified two of them as *Cynopterussphinx* and *Rhinolophusrouxii* (Figure 4). A third specimen was found along the highway we surveyed (7.724550°N and 81.212600°E) on August 9, 2015 (0822 h). External morphological characteristics (Table 2, Figure 5)– long tragus with a prominent notch, wing membrane attachment to the ankle, and short golden hair on forearms and hands– suggested that the specimen could belong to genus *Phoniscus* which is restricted to Southeast Asia and Australasia (Table 2). We named this specimen as Phoniscuscf.jagorii given its close resemblance to Peter’s trumpet-eared bat (*Phoniscusjagorii*) (Figure 5). Due to extensive damage to the facial structure, we could not make any detailed accounts on the dentition.

**Table 2.** Morphological characteristics and morphometric features (mm) of the road-killed specimen (Phoniscuscf.jagorii) from Peripheral areas of Maduru-Oya National Park,, *Phoniscusjagorii*, *Kerivoulahardwickii*, and *Kerivoulapicta* from Asia (Bates and Harrison 1997a; Blanford 1888-91; Corbet and Hill 1992; Dobson 1848–1895; Francis 2008a; Hill 1965; Phillips 1980; Tomes 1858).

Character	Unidentified road-kill specimen (Phoniscuscf.jagorii)	* Phoniscus jagorii *	* Kerivoula hardwickii *	* Kerivoula picta *
Muzzle	The facial structure is damaged beyond characterization	Extremity of the mussel is projecting; covered with hair	Covered with hair; moderately small. Short, and pointed	Moderately small; long and rather pointed; densely covered with long hair which overhangs the mouth
Ears	Large, few short hair at the base of ears; Tip of the ear rounded with a few short hairs (ear length: 14.03; ear width: 11.20)	Funnel–shaped large ears with rounded tips; two slight concavities– one just below the apex and another at the center of the posterior margin	Ears naked and relatively large, funnel shaped, tip rounded, inner margins regularly convex from base to tip, outer margins deeply concave immediately below the tip, the lower portions regularly convex. a prominent notch on tip of the posterior edge.	Moderately long; separate and distinctly funnel–shaped, bluntly pointed at tips; inner and outer margins terminating close together and giving the impression of almost complete cups; partially covered with short hair on the outer side
Tragus	Long, narrowing gradually to a point and a deep notch present on the posterior edge (tragus length: 8.44; tragus width: 2.11)	Tragus rather broad at base and tapers to an acute point; white with deep notch on posterior edge	Tragus very long, narrow, and attenuated, outer straight–sided and sharply pointed	Very long, slender, grey in color; terminating in fine points
Dorsal area of the body	Fur golden brown, black, and hair banded with four colors; dark grey–brown bases, then a buff band, then dark brown, then golden tips	Overall, golden brown and black; fur with four bands of color including a pale tip: dark brown or blackish–brown at the base, followed by buff, then brown, and finally golden or whitish–yellow tips; the paler tips are more pronounced on the ventral surface	Hair very soft and of moderate length; confined to the body and ceasing abruptly, both on the upper and the lower sides. General color of the upper parts rufescent brown although a few hairs could be pale–tipped; the hairs of the head, shoulders and mantle unicolored, those of the lower back and hinder parts generally, with the basal portions dark grey	Fur rather long, dense, and woolly; Extending slightly onto the membranes near the body; dorsal fur orange or tawny–red
Ventral area of the body	Fur paler grey to dark brown with golden tips	Under parts paler with slightly greyer tips	Lower parts, light rufescent–fawn, with the basal portions of the hairs dark brownish grey and paler grey hair tips; membranes and ears unicolored, semi–transparent blackish brown	Compared to dorsum; ventral hair is paler and yellowish
Ante–brachial membrane	Naked	Naked	Semi–transparent, thin in texture	Sparingly but visibly covered with many minute hairs on both surfaces; bright orange to scarlet in color.
Wing membrane	Naked and well developed. Attached to the ankle	Attached to the ankle	Arise from the base of the outer toes. Brown–colored but nearly transparent; upper surface almost naked, expect for a thin spread of small hair.	Moderately long and broad with the membrane attached to the base of the toes; orange to bright scarlet along the length of the forearms and fingers the rest is black
Interfemoral membrane	Ventral side naked, dorsal side is partly covered with hair; membrane is well developed and semi–transparent	Hair very short and almost invisible; the margins are mostly naked; sometimes with sparse sprinkle of hair	Naked, well developed; thin in texture and semi– transparent. Long scattered hair present on femur and tibia, feet are almost naked; no prominent fringe on the posterior boarder but scattering hair may be present	Sparingly but visibly covered with many minute hairs on both surfaces; bright orange to scarlet in color
Tail	Tip of the tail projects slightly (tail length: 40.54)	Tip of the tail projects slightly	Tail considerably shorter than the head and body	Long tail (as long as the head and the body) entirely contained within the interfemoral membrane
Radio–metacarpal pouch	Absent	Absent	Absent	Absent
Forearms and hand	The metacarpals and digits covered with short, golden hair (Forearm length: 37.64; thumb + claw length: 7.33; 2^nd^–5^th^ metacarpal lengths: 37.37, 36.80, 34.32, 35.04)	Short shiny yellow hairs along forearm and fingers	Hair absent on forearms or hands	No hair on forearms; Hair on metacarpals and digits are sparse and scattered. Bright orange color on all fingers and metacarpals
Feet	Few short golden hairs present (tibial length: 18.70; foot length: 09.58)	Short hair present on the hind feet	Feet small, equipped with relatively long, sharp claws, with a few short hairs on the toes	Feet small, densely–covered with short reddish–brown hairs and equipped with sharp small claws
Calcar	Long; covered with a dense fringe of short golden hairs (calcar length: 11.22)	Calcar relatively long; No hair on the calcar	Calcar long, extending approximately two–thirds of the distance from the ankle to the tail	Well developed with no lobes; covered with a dense fringe of short reddish hairs

### Selection of roosting sites and habitat associations

We noted four different types of roosting sites frequently occupied by bats in our study area. These included caves of different sizes, different locations of large mature trees and banana trees, abandoned buildings, and underneath of bridges (Table 1); all roosting sites were located within or in proximity to (140 m) dense forests or sparse woodlands. Fig. trees (*Ficus* spp.) were exclusively used by tree-roosting bats. Roosting trees had a dense canopy with no low-lying branches. The bats occupied branches approximately 3 m above the ground level. Roosting bats were not found inside home gardens, agricultural lands (with the exception of banana plantations), or open habitats such as grasslands or scrublands. Of the 16 active roosting sites we surveyed, seven of them were caves. Multiple species occupied a single type of roosting sites therefore roost selection may not be species-specific. For instance, three species occupied caves while another two species used abandoned buildings for roosting (Table 1). However, roost-sharing was only observed in two instances during our survey; *Megadermalyra* and *Hipposideroslankadiva* roosted underside of a concrete bridge (height 3 m from the streambed) over a slow-flowing stream with a dense forest cover along the banks while *Taphozouslongimanus* and *Rhinolophusbeddomei* roosted in the same cave located in a woodland habitat. For all four species, roost-sharing was only observed during the dry season.

**Figure 6.**
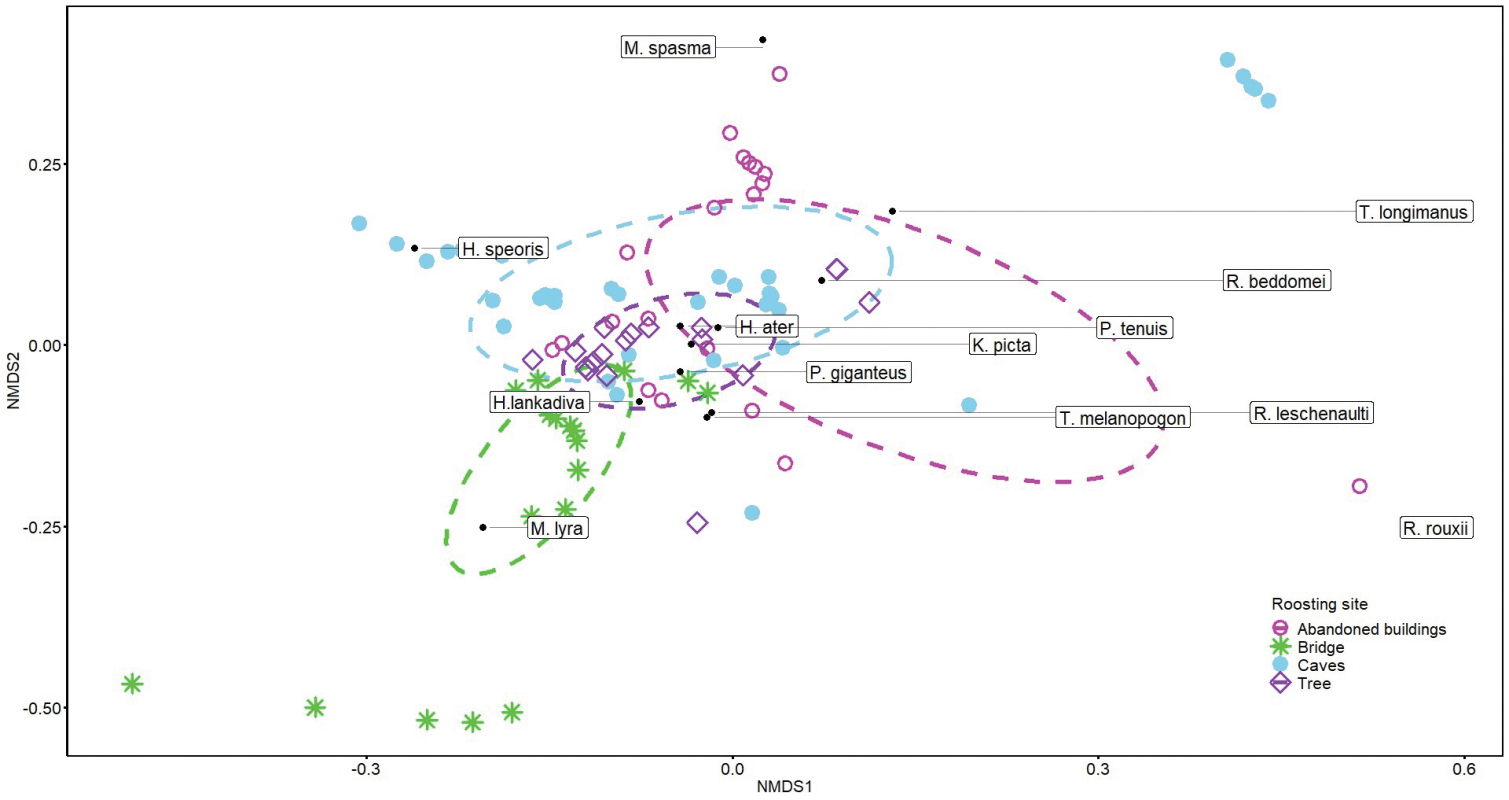
NMDS Ordination of Bat association with different roosting sites in Maduru-Oya National Park periphery. The ellipses represent 95% confident intervals around the centroids. *Cynopterussphinx* and Phoniscuscf.jagorii, which were only recorded as dead specimens, were not included in the ordination.

The MRPP analyses showed significant differences in species composition of bats across different roosting sites (Chance corrected within group agreement = 0.14, expected δ = 0.73, observed δ = 0.62, p = 0.001). The NMDS ordination reached a stable solution (mean stress = 0.08) after 500 iterations and produced two axes. The Monte Carlo randomization also suggested that the two axis solution was optimal (stress in randomized data = 0.26, p < 0.05). The stress level we reached (closer to 0.05 and less than 0.1) in our stable solution with two dimensions suggests a good fit of our dissimilarity object. The ordination plots (axes 1 and 2) indicated substantial segregation of the different species based on the roosting sites selected (Figure 6). *Megadermalyra* and *Hipposideroslankadiva* ordinated exclusively with underside of bridges. *Rousettusleschenaulti*, *Rhinolophusrouxii*, *Hipposiderosater*, and *Megadermaspasma* ordinated in close association with the abandoned buildings (although some of them ordinated outside the 95% confidence interval). *Hipposiderosspeoris*, *Rhinolophusbeddomei*, *Taphozouslongimanus*, and *Taphozousmelanopogon* closely ordinated with the caves. *Pipistrellustenuis, Pteropusgiganteus*, and *Kerivoulapicta*, ordinated with trees. However, 95% confident ellipses for roosting locations overlapped substantially.

The GAM we constructed indicated that both the percent cover of crop-mixed natural vegetation and percent forest cover at 500 m radius are of significantly important predictors of bat presence in potential roosting sites (Table 3; adjusted model correlation coefficient = 0.412; deviance explained = 45.3%). Although percent cover of agricultural lands within a 5 km radius had a relatively greater coefficient estimator, its impact was marginally insignificant.

**Table 3.** Local (500 m radius around the roosting site) and landscape-scale (5 km radius around the roosting site) predictors of bat presence at potential roosting sites derived from a binomial generalized additive model (no large wetlands or aquatic land cover types were found within a 500 m radius).

Land-use variable	Coefficient estimate		z		p	
	500 m	5 km	500 m	5 km	500 m	5 km
Agricultural lands	0.52	-3.10	0.97	-1.30	0.33	0.19
Scrublands	-0.32	0.47	-0.96	0.49	0.34	0.62
Crop-mixed scrublands & woodlands	0.60	-0.84	2.10	-0.90	0.04*	0.37
Other vegetation mosaics	0.75	-0.71	1.54	-0.87	0.12	0.39
Forests (woody vegetation)	1.38	-0.67	2.81	-0.76	0.005 **	0.44
Wetlands and other open water bodies	n/a	0.13	n/a	0.22	n/a	0.82

## Discussion

The bat species richness in the peripheral areas of Maduru-Oya National Park is remarkable as this bat community represents six of the seven Sri Lankan chiropteran families and ~50% of the island’s total bat species (Bates and Harrison 1997a, Yapa 2017). The species richness we documented is comparable to or greater than the diversity recorded from other similar regions of Sri Lanka. For instance, baseline biodiversity surveys at Wasgamuwa National Park (located in the same bioclimatic region as Maduru-Oya area) documented seven species of bats (Department of Wildlife Conservation 2007). Kusuminda et al. (2013) reported six species of bats in a Buddhist monastery which encompassed extensive secondary forests with numerous caves. In the intermediate zone of Sri Lanka, Yapa (1991) found five bat species in a cave complex. However, compared to other tropical roosts, colony sizes we reported were remarkably low (100,000–500,000 cf. ~300) (Rubsamen et al. 2004; Yapa 1992). Our survey also confirmed presence of several rare and threatened bat species outside protected areas in Sri Lanka (*Hipposideroslankadiva*, *Kerivoulapicta*, *Taphozouslongimanus*, *T.melanopogon* and *Rhinolophusbeddomei*). Thus, our study calls for science informed management and conservation of habitats around Maduru-Oya National Park.

Our discovery of Phoniscuscf.jagorii in our survey is noteworthy. The genus *Phoniscus* is currently known from four species, three of which are from Southeast Asia, New Guinea, and Eastern coast of Australia (Corbet and Hill 1992; Hutson et al. 2001; Wilson and Reeder 2005). This genus has not so far been recorded from Indian subcontinent or nearby south-Asian islands, thus our observation might be the first documentation of the genus *Phoniscus* for South Asia. *Phoniscus* species are forest dependent (found in both primary and secondary forests) and are found mostly in lowland rainforests, dry dipterocarp forests, and semi-evergreen forests (Francis 2008b). The closest species match to the specimen we found – *Phoniscusjagorii* (Peter’s Trumpet-eared Bat)– have been documented throughout Southeast Asia, including both the mainland and the Malayan archipelago: Bali, Borneo, Java, Laos, Lombok, Peninsular Malaysia, Samar, South China, South East Asia, Sulawesi, Thailand, and Vietnam (Beolens et al. 2009; Francis 2008b; Thong et al. 2006). *Phoniscusjagorii* is a low-flyer (Thong et al. 2006), thus could be susceptible to vehicular collisions, which supports our documentation. The damage sustained by the facial structure of our specimen precluded proper species identity. Given the geographical disjunction between Sri Lanka and *Phoniscus* biogeography, our specimen could represent a new species (Pipat Soisook, [Prince of Songkla University, Hat Yai, Songkhla, Thailand] and Chelmala Srinivasulu [Osmania University, Hyderabad, India] pers. comm. [September 2017]). We recommend further sampling via mist-netting, bioacoustic surveys, active surveys in roosting sites, eDNA-surveys, and molecular phylogenetic analyses to confirm the taxonomic status of this specimen (Walker et al. 2016).

Nearly all the active roosting sites we documented were located within or in proximity to dense forests. These observations suggested that most bats of Maduru-Oya area are forest dependent. Our GAM for species presence also indicated forest dependency of these bats in terms of roost selection. Forest habitats provide a diverse array of high-caloric and nutrient-rich food resources for foraging bats. Our inferences about forest-dependency of bats are consistent with surveys conducted elsewhere in Sri Lanka and other humid tropical regions (Furey et al. 2010; Neuweiler et al. 1987; Phillips 1980). However, the forested habitats outside Maduru-Oya National Park are fragmented into isolated, smaller patches. Studies on Palaeotropical forests have shown that bats roosting in tree cavities and foliage can be more susceptible to habitat fragmentation due to loss of suitable roosting sites and negative consequences of edge effect (Struebig et al. 2008). Furthermore, most insectivore bats we recorded in our study are narrow-space foraging guilds that possess ecomorphological specializations (wing dimensions) and echolocation signals to forage in high-clutter forest interiors; such guilds can be affected by reduced availability of foraging areas as well as edge effect (Kingston 2010; Lane et al. 2006; Struebig et al. 2010).

Although we visited >50 potential roosting sites, only a quarter of those had bats at least once during our surveys. Also, a number of caves we visited had indirect evidence (guano) for bat presence. These observations may indicate lower site fidelity of bats at our study area where bats may shift between different roosting sites. Such high roost lability can be attributed to lower cost in commuting to foraging grounds, high familiarity with multiple roosts that vary in microclimatic conditions, greater availability of high-quality roosts; nest lability also ensures increased cross breeding potential, reduced predation risk, and lowered parasitic loads (Lewis 1995). Besides, bats change roost location based on their life-history stage (pregnancy vs. lactation vs. post-lactation), which is particularly common among female bats (Lausen and Barclay 2002). Alternatively, low-roost occupancy we documented (only 52% of the roosting sites surveyed had either direction or indirect evidence for bat occupancy) can be an artifact of high selectivity over roosting sites. Bats have high preference to roosting sites with multiple entry points, greater complexity of the roost interior, larger roosting area, broader interior temperature range, lack of anthropogenic disturbances both inside and outside the roosting site (Boyles 2007; Phelps et al. 2016). Moreover, the extent of modified land-cover types at both local and landscape-level around the roosting site can also influence the species composition of bats of a given roosting site.

Access to suitable roosting sites is a critical element for bats’ life-history functions. Human-occupied landscapes usually contains forest preserves, mature woody vegetation, and buildings, therefore, may provide shelters for bat roosting compared to agricultural landscapes under intensive commercial farming which may lack a diverse array of roosting sites (Gehrt and Chelsvig 2003). Microclimatic stability and thermoregulatory advantages, protection from inclement weather and predators, nursing young, and grooming are some benefits conferred from roosting sites; social behaviors, such as determination of hierarchies, competition, cooperation, and recruiting females into “harems” are also critical ethological elements of roost selection (Campbell et al. 2006; Chaverri and Kunz 2010; Lewis 1995; Schmidt et al. 2011; Storz and Kunz 1999; Tan et al. 1997). Selection of optimal roost have profound fitness consequences for bats as roost conditions cater to their diversified functional attributes, physiological optima, life-history specifications, and social integrity (Campbell et al. 2006).

Although our survey covered potential roosts in agricultural habitats, none of those were occupied by bats, except for *Kerivoulapicta* we documented from a banana plantation. Reduced overall bat activities (foraging and roosting) have been reported from agricultural landscapes in tropical Southeast Asia as well as the northern template of American Midwest (Gehrt and Chelsvig 2003). Lack of dense tree cover, monotypic vegetation, pesticide use, prey scarcity, intensive crop management activities, and limited access to water may have rendered agricultural habitats unsuitable for bats (Gehrt and Chelsvig 2003). Although we did not find active bat roosts in proximity to human settlements, our overall study area has substantial human occupancy and built-up environments, yet, provides suitable roosts for bats. Similarly, remarkably high bat activities have been documented in human-inhabited urban and suburban landscapes with sizable woodlots with suitable roosting sites (such as large mature trees, less-used buildings), reliable water sources, and suitable foraging grounds such as woodland edges and urban parks (Gaisler et al. 1998; Gehrt and Chelsvig 2003; Sparks et al. 2005).

Caves appeared to be the preferred roosting sites for most bats in our study area; similar habitat preferences have also been reported elsewhere in Sri Lanka (Yapa and Ratnasooriya 2006; Yapa et al. 2011; Yapa 1992) as well as throughout the Indo-Malayan realm, especially in the karst ecosystems of southeastern Asia (Furey et al. 2010). Caves serve as microclimatically-stable, predator-safe roosting habitats for both adults and juveniles (Chaverri and Kunz 2010; Kunz and Lumsden 2003; Yapa et al. 2011). Being endotherms, occupying thermally optimal environments yields bats with energetically-efficient metabolism. In dry tropical environments, bats use caves for aestivation and in the temperate zone for hibernation (Lewis 1995). In monsoon-dependent dry zone of Sri Lanka, water is a limited resource as most of the surface waters are ephemeral. However, some of the bat-occupied caves we surveyed provide year-round access to water making those caves suitable roosting sites for bats. Our observations on roost-sharing was limited to two instances (*Hipposideroslankadiva* and *Megadermalyra* underneath a bridge and *Taphozouslongimanus* and *Rhinolophusbeddomei* in the same cave). In stark contrast, roost-sharing has been frequently observed throughout both the Old World and the New World (Bates and Harrison 1997b; Eckrich and Neuweiler 1988; Rubsamen et al. 2004). Greater availability of suitable roosting sites in Maduru-Oya area may have negated the need for sharing refugia.

Members of the family Pteropodidae (Old World Fruit bats) mostly roost on large, mature trees and deserted buildings as confirmed by our study (Chaverri and Kunz 2010). They also exhibit a wide variation in roosting sites including foliage of large-leaved trees, dead or dry palm fronds, seed strings, in bark recesses, and aerial roots (Campbell et al. 2006; Digana et al. 2003; Kunz and Lumsden 2003; Storz and Kunz 1999; Tan et al. 1997) although our survey did not reveal comparable observations. Being predominately frugivorous, roosting on large fruiting trees may provide easy access to food for pteropodid bats. Dense canopy and large, well-grown branches of mature trees also provide stable roosting substrates and protection from predators. Moreover, roost selection of some Old World fruit bats is biased towards certain tree species and prefers riparian trees in undisturbed forests (Mildenstein et al. 2005). Although tent constriction have been observed among tree-roosting bats, we made no such observations in our survey (Chaverri and Kunz 2010; Digana et al. 2003). Roosting sites we surveyed were mostly used as day roosts indicating extensive nocturnal activity which also agrees with general activity patterns of tropical bats that have lengthy daily activities from dawn to dusk (Yapa et al. 2011).

Presence of bats in potential roosting sites was only significantly influenced by forest cover and crop-mixed natural vegetation cover (woodland and scrubland mosaics with multiple types of crops) whereas the former was the most impactful predictor. Both of those predictors were only significant at local (500 m radius around the roosting site) scale not at the landscape-scale. Importance of mixed vegetation mosaics, particularly those embedded with polyculture practices (comparable to analog forests and permaculture systems) as a local-scale land-cover predictor warrant further investigation. This may indicate use of such vegetation mosaics by open-space and edge foragers. In contrast to our findings, a multitude of other studies have underscored the importance of landscape-scale features for bat occupancy in potential roosting sites (Gaisler et al. 1998; Gehrt and Chelsvig 2003; Sparks et al. 2005). Our study area was predominantly covered by forests at landscape scale, thus, forest cover across a 5 km radius may have been less variable among different roosting sites.

### Conservation challenges and recommendations

Mahaweli Development scheme and subsequent expansion of human settlements, and agricultural intensification have resulted in tremendous habitat transformations in the landscape structure in Maduru-Oya area (Ekayanake 1987; Manatunge et al. 2008). Such socioeconomic schemes resulted in several novel anthropogenic disturbances, such as felling large trees for lumber, burning grasslands for livestock, reduced tree cover, lack of habitat connectivity, and agricultural expansion which are detrimental for local bat fauna (Furey et al. 2010; Gaikwad et al. 2012; Mickleburgh et al. 2002; Mildenstein et al. 2005). Moreover, slash-and-burn farming destroys lower vegetation cover and fire consumes snags, tree cavities, and cluttered foraging grounds (Hutson et al. 2001; Mickleburgh et al. 2002). In the recent decades, application of broadcast pesticides for vector control has substantially increased in Maduru-Oya area (Amerasinghe et al. 1991), which can impact the bats’ prey base (Mickleburgh et al. 2002; Weerakoon and Goonatilake 2006). Snag removal and demolition of alternative roosting sites (abandoned huts, barns, and mines) are also detrimental for bats (Hutson et al. 2001; Lane et al. 2006). Folklores combined with perceived fear of diseases may lead to vengeful killing in our study area (Dickman and Hazzah 2016; Klimpel and Mehlhorn 2016). Pteropodids are considered pests by fruit farmers, thus are targeted for extermination in our study area. Large bats have long been exploited as bush meat in Indian oceanic islands, which was consistent with our occasional observations at Madudu-Oya National Park periphery (Mickleburgh et al. 2009; Mickleburgh et al. 2002).

We recommend a landscape-scale approach for bat conservation of Maduru-Oya area, which entails conservation of roosting sites and associated habitats, particularly forest patches and aquatic habitats both within the national park and peripheral wilderness (Avila-Flores and Fenton 2005; Gehrt and Chelsvig 2003; Jaberg and Guisan 2001). Bat roosting sites also provide habitats for other species, such as snakes, geckos, rodents, toads, and invertebrates. Cave systems in our study area also have cultural, aesthetic, historical, paleontological, and geological importance, thus conservation of these caves also confers multitude of benefits (Kingston 2010; Mickleburgh et al. 2002). We propose adoption of wildlife-friendly land management practices in Maduru-Oya peripheral areas. Although much of our study area is considered the buffer zone of the park, neither management actions nor legislative enforcements are implemented there (Department of Wildlife Conservation 2004). We suggest zonation of land uses and limiting human activities in and around bat roosting sites. Introduction of organic farming and permaculture might minimize agrochemical use while increasing the habitat heterogeneity of the buffer zone. Agroforestry systems that include forest gardening, alley cropping, intercropped fruit and nut bearing trees for shade and folder, forested riparian buffers, and tree-planted hedgerows as windbreaks will make agro-pastoral systems amicable for bats since such landscape elements are critical for bat activities (Harvey and Villalobos 2007; Hochegger 1998; Jacob and Alles 1987; Long and Nair 1999). Bats have high fidelity to landscapes with multiple suitable roosting sites (Avila-Flores and Fenton 2005; Lewis 1995). Thus, maintaining redundancy in suitable roosting sites provides insurance against loss of primary roosts (Mager and Nelson 2001).

Different species of bats we documented differed markedly in their natural histories. For instance, *Rhinolophusrouxii* forages in the foliage of dense forests (Phillips 1980; Rubsamen et al. 2004) while *Hipposiderosspeoris* is equipped to forage in dense scrublands, woodlands, river channels, and wetlands (Bates and Harrison 1997a; Pavey et al. 2001). Furthermore, critical resources required for bats are distributed throughout the landscape, and these resource demands vary seasonally, among variable life-history stages, and between sexes (Broders and Forbes 2004; Jaberg and Guisan 2001). Thus, conservation of Maduru-Oya bats should focus on managing a mosaic of interconnected habitats including forest patches, lake fringes, wetlands, river channels, and riparian buffers. Aquatic habitats provide profitable food resources (high-density insect swarms); forest patches provide suitable roosting sites while forested landscapes are used as refuge against predators (Broders and Forbes 2004; Jaberg and Guisan 2001). Woodlots of different vegetation types, snags of variable decay classes, caves of different sizes, and isolated mature trees should be systematically protected.

Our study underscored the importance of conservation outside protected areas. Previous studies have also highlighted the importance of “trees outside the forests” for biodiversity conservation (Long and Nair 1999). Maduru-Oya National Park periphery is dominated by secondary vegetation types ranging from scrublands to seasonal mixed-deciduous evergreen forests, yet, supported a diverse assemblage of bats. Importance of such novel ecosystems for overall biodiversity conservation in Sri Lanka is not trivial (Pethiyagoda 2012; Pethiyagoda and Manamendra-Arachchi 2012). Since our study was focused synanthropic bat communities, we hope that our study provides a foundation for exploring conservation potential in anthropocentric environments.

Chiropterans are salient for multiple ecological functions, regulating invertebrate populations, serving as a prey-base, seed dispersal, and pollination, thus conservation of bats is imperative for healthy ecosystems (Digana et al. 2000; Eckrich and Neuweiler 1988; Jayasekara et al. 2003; Medellín et al. 2000; Neuweiler et al. 1987). Agriculture is the main livelihood of Maduru-Oya area where insectivorous bats can keep agricultural pests in check and effectively regulate medically important pests such as mosquitos. Given their critical ecosystem services, bats can be considered as both a keystone species (especially in cave and subterranean ecosystems) and an umbrella species that ensure conservation of whole landscapes (Mildenstein et al. 2005; Phelps et al. 2016). Our findings will also contribute towards developing species distribution maps, Red List assessments, conservation prioritizations, and influence local land management around Maduru-Oya National Park.
